# Polyol-mediated zinc oxide nanoparticles using the refluxing method as an efficient photocatalytic and antimicrobial agent

**DOI:** 10.3389/fbioe.2023.1177981

**Published:** 2023-04-19

**Authors:** Payal Walunj, Arpita Roy, Vikram Jadhav, Pragati Athare, Akshay Dhaygude, Jayraj Aher, Jari S. Algethami, Dnyaneshwar Lokhande, Mohammed S. Alqahtani, Arun Bhagare, Saad Alghamdi, Lienda Bashier Eltayeb, Issa Saad Al-Moraya, Krishna Kumar Yadav, Yongtae Ahn, Byong-Hun Jeon

**Affiliations:** ^1^ M. V. P. Samaj’s K. K. Wagh Arts, Science, and Commerce College, Pimpalgaon (B.), Nashik, India; ^2^ Department of Biotechnology, Sharda School of Engineering and Technology, Sharda University, Greater Noida, India; ^3^ Department of Chemistry, K. R. T. Arts, B. H. Commerce, and A. M. Science College, Nashik, India; ^4^ Department of Chemistry, College of Science and Arts, Najran University, Najran, Saudi Arabia; ^5^ Promising Centre for Sensors and Electronic Devices (PCSED), Najran University, Najran, Saudi Arabia; ^6^ Department of Chemistry, M.V.P. Samaj’s K.P.G. Arts, Science, and Commerce College, Igatpuri, Nashik, India; ^7^ Radiological Sciences Department, College of Applied Medical Sciences, King Khalid University, Abha, Saudi Arabia; ^8^ BioImaging Unit, Space Research Centre, University of Leicester, Leicester, United Kingdom; ^9^ Laboratory Medicine Department, Faculty of Applied Medical Sciences, Umm Al-Qura University, Makkah, Saudi Arabia; ^10^ Department of Medical Laboratory Sciences, College of Applied Medical Sciences, Prince Sattam Bin AbdulAziz University- Al-Kharj, Riyadh, Saudi Arabia; ^11^ Forensic Medicine Center, Ministry of Health, Saudi Arabia; ^12^ Faculty of Science and Technology, Madhyanchal Professional University, Bhopal, India; ^13^ Environmental and Atmospheric Sciences Research Group, Scientific Research Center, Al-Ayen University, Thi-Qar, Iraq; ^14^ Department of Earth Resources and Environmental Engineering, Hanyang University, Seoul, Republic of Korea

**Keywords:** antimicrobial activity, photocatalytic activity, refluxing method, ZnO NPs, polyol

## Abstract

Nanomaterials have attracted more curiosity recently because of their wide-ranging application in environmental remediation and electronic devices. The current study focuses on zinc oxide nanoparticles’ (ZnO NPs) simple production, characterization, and applications in several fields, including medicinal and photocatalytic degradation of dyes. The non-aqueous-based reflux method is helpful for ZnO NP synthesis; the procedure involves refluxing zinc acetate dihydrate precursor in ethylene glycol for 3 hours in the absence of sodium acetate, in which the refluxing rate and the cooling rate are optimized to get the desired phase, and the unique morphology of polyol-mediated ZnO NPs; it has been achieved using the capping agent TBAB (tetra-butyl ammonium bromide) and precursor zinc acetate dihydrate. UV–Vis, FTIR, XRD, and FESEM structurally characterized polyol-mediated ZnO-NPs. The results show that the material is pure and broadly aggregated into spherical nanoparticles with an average particle size of 18.09 nm. According to XRD analysis, heat annealing made the crystallites more prominent and favored a monocrystalline state. These results and the low cost of making polyol-mediated ZnO NPs demonstrate photocatalytic and antimicrobial properties.

## 1 Introduction

For technology to work in the twenty-first century, nanoscale-sized materials must be functionalized; the scientific field of nanostructures includes manipulating materials at the nanoscale. Because of their large, unique area and surface potential, and distinctive surface characteristics, nanomaterials behave like atoms. These nanoscale materials compare bulk materials showing higher surface-to-volume ratios than bulk materials. Larger particles’ physical properties are more stable, have lower surface-to-volume ratios, and have a narrower range of applications. When altered at the nanoscale level, bulk materials show enhanced and distinctive properties due to their size, shape, and morphology (Khan et al., 2022). They are helpful in various fields, such as material science, photocatalysis, medicine, and semiconducting material (Michael et al., 2022). Significant research on heterogeneous photocatalysis over semiconductor oxides for various environmental and energy-related applications is underway. This method mainly addresses ecological problems, such as the degradation of several air, water, and soil pollutants, and biological activities, such as antibacterial, antifungal, and anticancer properties (Dhayagude et al., 2017; Roy et al., 2022a; Pandit et al., 2022). So far, many metal oxide nanoparticles have been investigated for their biological and photocatalytic potential, including the various metal oxides (Pandit et al., 2022), and metal sulfide semiconductors have been studied for their biological potential and catalytic activity to reduce pollution (Jadhav et al., 2022a).

Moreover, nanomaterials have also demonstrated potential applications in electronics and energy storage. The unique electronic properties of nanoparticles allow for the synthesis of new materials with enhanced electrical conductivity, increased surface area, and improved mechanical properties (Hong et al., 2009). For instance, carbon nanotubes have been used to develop high-performance energy storage devices such as supercapacitors and batteries. The use of nanomaterials in the biomedical field has gained considerable attention in recent years. The small size of nanoparticles enables them to penetrate biological barriers and interact with cells and tissues, making them ideal candidates for drug delivery and imaging applications (Roy et al., 2021). Nanomaterials have been used for tissue engineering and regenerative medicine applications. Recently, researchers have developed nanofibrous scaffolds to guide tissue regeneration and stimulate cell growth (Mirzaei and Darroudi, 2017). The unique properties of nanomaterials have made them attractive candidates for a wide range of applications in various fields (Roy et al., 2022b). With ongoing research and development, the potential for nanotechnology to revolutionize multiple industries and address significant societal challenges is promising. However, the potential risks of using nanomaterials, such as toxicity and environmental impacts, must also be carefully evaluated and addressed (Kumar et al., 2013).

**SCHEME 1:**
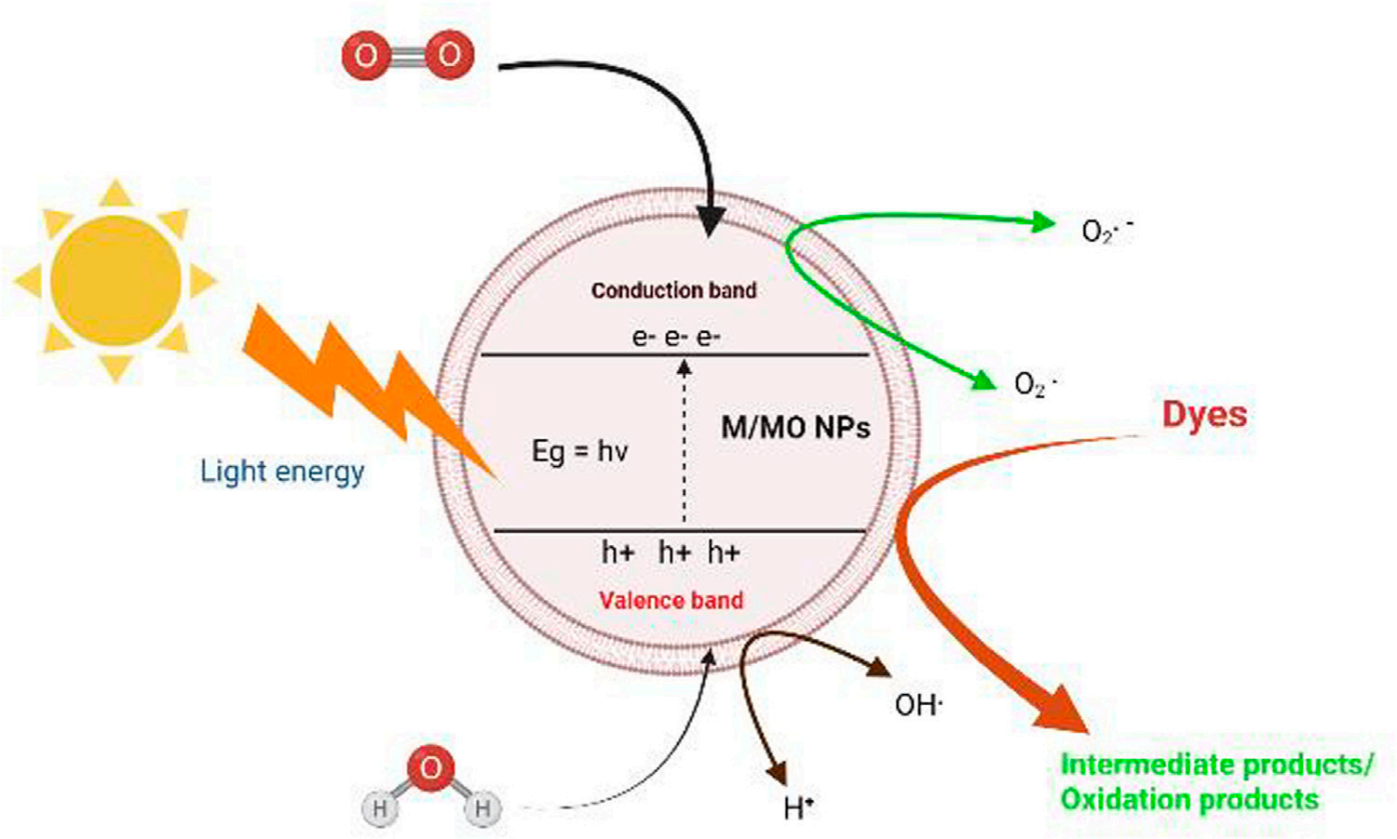
Process of photocatalytic dyes degradation (metal/metal oxide nanoparticles).

Due to the more significant reducing and oxidizing power of photogenerated electrons and holes, comparatively cheap cost, chemical and biological stability, and non-toxicity, TiO_2_ is the most investigated photocatalyst among the many explored semiconductors. To improve the photocatalytic performance of TiO_2_, much more effort has been made, including using various chemical processes, transition metal doping, anion doping, linked catalysts, and noble metals (Gupta and Tripathi, 2011). Advanced reaction approaches include the photocatalytic treatment of chemical contaminants using semiconductors as photocatalysts. Numerous research studies on a semiconductor such as TiO_2_ photocatalytic activity have been published. TiO_2_ and most other photocatalysts show low responsiveness to UV irradiation, which consumes 4% of solar energy, severely restricting their wide-ranging practical use. The usage of TiO_2_ is greatly hampered since direct sunlight generally comprises just 4% of UV light, compared to visible light’s 43% limitless solar energy. The unique uses of ZnO in optics, optoelectronics, catalysis, pyro-electricity, and piezo-electricity have recently become evident (Nagaraju et al., 2020). Because of their superior oxidation property, ZnO NPs have the highest photocatalytic activity of all the inorganic photocatalytic materials (Dhayagude et al., 2017). They frequently remove environmental contaminants, including dyes, poisons, and pigments (Jiang et al., 2018). There is a process through which the impurity is entirely degraded. The electrons from the valence band (VB) immediately become excited and go to the conduction band (CB) when exposed to UV radiation. ZnO is an n-type semiconductor with a significant exciton binding energy of 60 meV and a bandgap of about 3.37 eV. Due to the VB, empty sites produce electron-hole pairs. As a result, electrons collect in the CB, whereas holes accumulate in the VB. These holes in the band’s valence create hydroxyl groups when they interact with water molecules, which then combine with the dye to make the degraded products, along with CO_2(g)_ and H_2_O_(g)_, as shown in Scheme 1. Oxygen reduction to O_2_ due to an interaction between oxygen atoms and CB electrons can also cause deterioration (Ong et al., 2018).

The use of nanoparticles in wastewater treatment is a promising area of research that has gained significant attention in recent years. One advantage of using nanoparticles is their large surface area, which enables efficient adsorption of contaminants from wastewater; nanoparticles can be functionalized to exhibit photocatalytic properties, allowing for the degradation of organic pollutants and the disinfection of wastewater (Wan et al., 2013). According to recent studies, wastewater treatment using nanoparticles was highly effective. In the past, several ecologically friendly techniques for the surface acting and photocatalytic degradation of contaminants in wastewater have been documented, including using zinc oxide nanoparticles in a surface layer of clay used as a catalyst. For the investigation in the current study using polyol-mediated ZnO, a model pollutant called Rhodamine-B, was used, and the photocatalytic activity of the dye was assessed as a percentage and its antimicrobial properties were studied. The nanoparticles can work against various bacteria, including *Escherichia coli*, and are a suitable antibiotic substitute. According to several research studies, the generation of reactive species such as O_2_^−^, OH^.^, and H_2_O_2_ action toward the antibacterial and antifungal activities in polyol-mediated ZnO nanoparticles (Sirelkhatim et al., 2015; Abdelmigid et al., 2022). Another effect of producing Zn^2+^ ions is the death of bacteria by causing damage to membrane proteins and the breakdown of cell membranes (Islam et al., 2022). This makes them a promising alternative to traditional antibiotics, which can lead to the development of antibiotic-resistant strains of bacteria (Shi et al., 2014). The current study will cover the synthesis of polyol-mediated ZnO NPs using the refluxing method, its characterization and photocatalytic activity of dye Rhodamine-B, and antibacterial and antifungal activities (Vaseem et al., 2010; Rahman et al., 2013; Wan et al., 2013; Shi et al., 2014; Sirelkhatim et al., 2015; Wojnarowicz et al., 2016; Soni et al., 2018; Roy et al., 2021; Roy et al., 2022b). The use of polyol-mediated ZnO nanoparticles in wastewater treatment shows great promise for developing efficient and sustainable methods for removing contaminants and disinfection of wastewater. Further research is needed to optimize the synthesis of these nanoparticles and evaluate their long-term environmental impacts (Abel et al., 2021).

## 2 Materials and methods

### 2.1 Materials

Zinc acetate dihydrate [**Zn(CH**_**3**_ **COO)**_**2**_ **.2H**_**2**_ **O, 99% AR**], tetra butyl ammonium bromide (TBAB) (C_16_H_36_BrN) (capping agent), Rhodamine-B (C_28_H_31_ClN_2_O_3_) (dye), ethanol, and ethylene glycol of analytical grades with good purity were used.

### 2.2 Preparation of solutions

The molar solutions were prepared in ethylene glycol using the standard method; 0.1 M dihydrated zinc acetate, 0.01 M TBAB, and 10^–5^ M Rhodamine-B (deionized water) (Jadhav, 2019).

### 2.3 Synthesis of polyol-mediated ZnO NPs using the refluxing method

**SCHEME 2:**
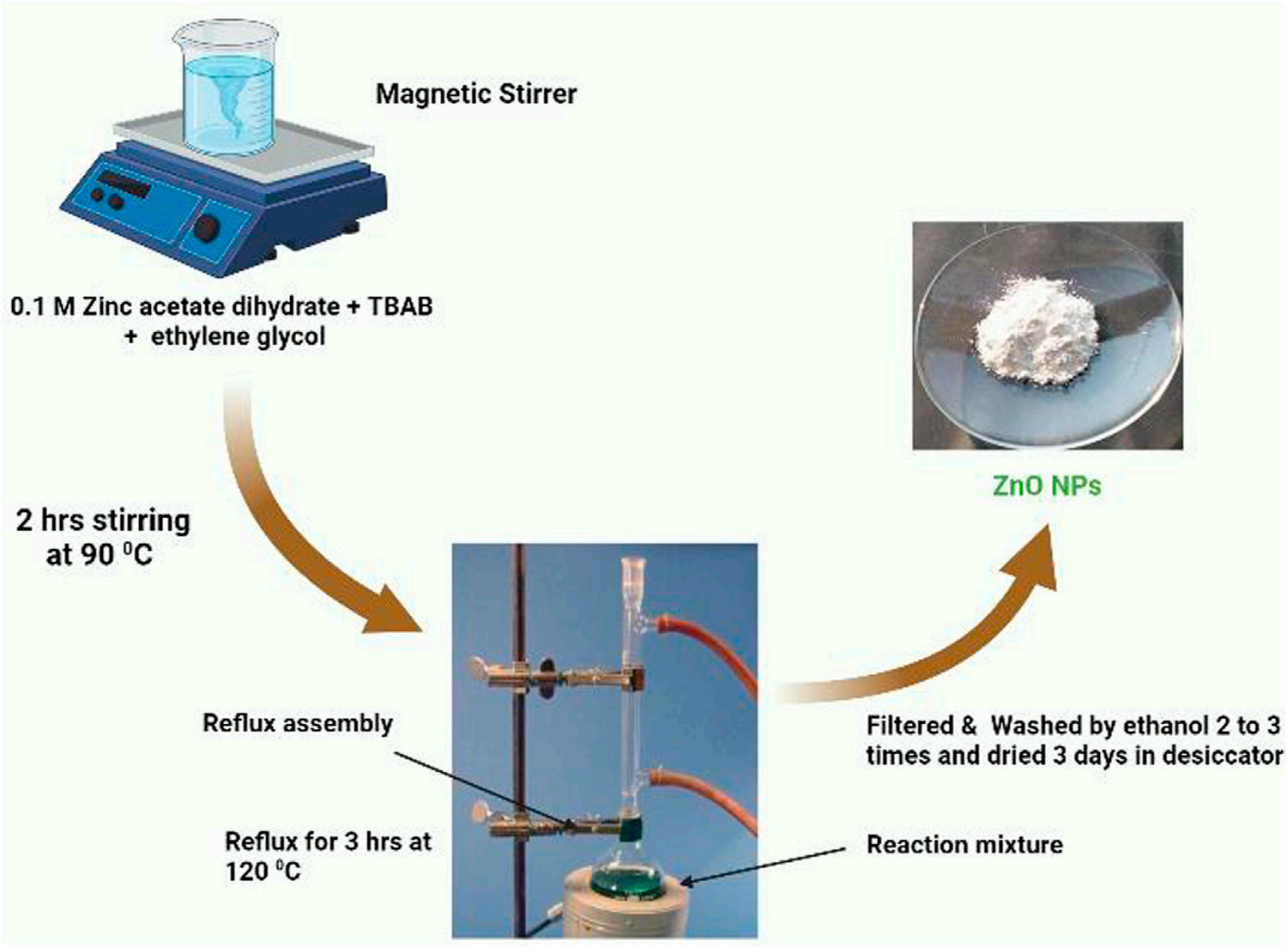
The synthesis process of polyol-mediated ZnO NPs.

0.1 M dihydrated zinc acetate and 0.01 M TBAB mixed with 60 mL ethylene glycol solution was stirred for a few minutes at 90°C using a magnetic stirrer, and this solution was added to a three-neck glass flask; it was heated with stirring for 3 h at 90°C, and the white precipitation started within 1 hour. The complete zinc oxide is formed when the bathing temperature is above 120°C. The reaction mixture was filtered, washed with ethanol, and centrifuged 2–3 times. The washed precipitate was dried in a desiccator for 3 days, removing moisture and protecting from water vapor in the air, and the polyol-mediated ZnO NPs were formed, as shown in Scheme 2.

The following chemical reaction describes the synthesis of polyol-mediated ZnO NPs using ethylene glycol;

$$Zn{\left({CH}_{3}COO\right)}_{2}\;.{2H}_{2}O+{HOCH}_{2}\;{CH}_{2}OH\;\frac{TBAB}{T\;and\;P}>\;{ZnO}_{\left(s\right)}+other\;products\;\left(liquid\;\&\;gas\right).$$

## 3 Results and discussion

### 3.1 UV–vis spectra

**FIGURE 1:**
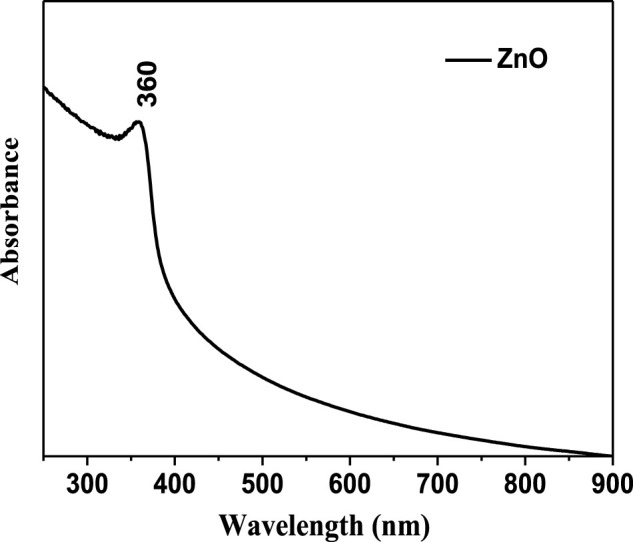
UV-Vis spectra of polyol-mediated ZnO NPs.

The absorption spectra of zinc oxide nanoparticles show a maximum absorption peak at 360 nm, as shown in Figure 1. The solid polyol-mediated zinc oxide spectra show substantial excitation binding energy at room temperature, offering good absorption capacity in the UV–vis region. According to the wavelength of the 360-nm absorption peak, the quantum confinement effect causes the ZnO NP absorption spectra to be blue shifted concerning its bulk value (377 nm), which is in excellent accord with prior findings. Bandgap energy generally decreases with increasing particle size.

The band gap was calculated using the following equation,

$$\begin{array}{c} Band\;gap=12400/\lambda \;in\;A^{0}\\ =12400/3600\\ =3.44\;eV. \end{array}$$

The standard band gap of ZnO NPs is 3.2–3.5 eV.

The calculated band gap of the synthesized ZnO NPs is 3.44 eV, which is within the range of the standard band gap for ZnO NPs (3.2–3.5 eV). The band gap energy is an important parameter in determining the optical properties of nanoparticles and plays a crucial role in applications such as photocatalysis and solar cells. The bandgap energy of ZnO NPs generally decreases with increasing particle size due to quantum confinement effects and surface states. The absorption spectra and calculated band gap of the synthesized ZnO NPs suggest that they possess good optical properties, which can be useful for various applications in catalysis, sensing, and energy conversion.

### 3.2 FTIR analysis

**FIGURE 2:**
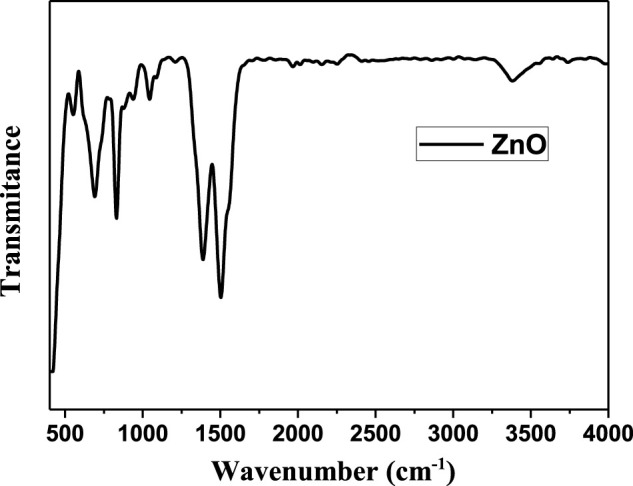
FTIR spectrum of polyol-mediated ZnO NPs.

FTIR measurements on the polyol-mediated zinc oxide nanoparticles were carried out at 25°C with the KBr method in the wavenumber region (500–4,000 cm^-1^). Studies on composition and purity of the zinc oxide nanoparticles were carried out using infrared technology. At 570–680 cm^−1^, the ZnO bond bending vibration peaks, as shown in Figure 2, indicate a low-energy region; the zinc complex shows the stretching band at 1,500 cm^-1^ and also may suggest that the N–H bending mode appears as a medium to the strong-intensity band in the range from 1,640 to 1,560 cm^−1^. The band shifts to a lower frequency in secondary amines and appears near 1,500 cm^−1^. The fingerprint region (below 1,000 cm^-1^) is significant for metal oxides, as it exhibits an absorption band caused by interatomic vibrations. The ZnO peak in this region (500–600 cm^-1)^ confirms the presence of zinc oxide in the nanoparticles. The peak observed in the higher energy region (3,600–3,450 cm^-1^) is due to the stretching vibration of the -OH group, and the peaks at 3,452.30 and 1,119.15 cm^-1^ may indicate O-H stretching vibration. Peaks at 1,604.0 and 620.93 cm^-1^ are due to stretching and deformation of ZnO, respectively. The influential bands seen at (1,120–1,065) cm^−1^ C–O stretching vibration may indicate the functional groups of alcohols and a carbonyl group.

### 3.3 X-ray diffraction microscopy

**FIGURE 3:**
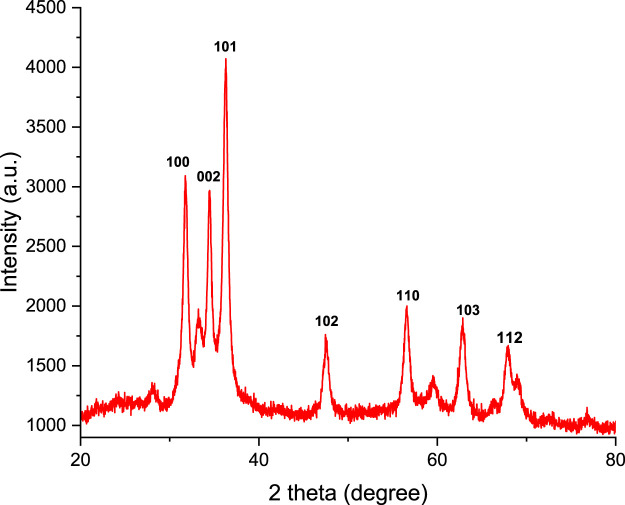
XRD spectra of polyol-mediated ZnO NPs.

The XRD spectra examining the crystalline phases in the polyol-mediated ZnO NPs powder are shown in Figure 3, and the prominent, strong peak there confirmed the synthesis of nanosized crystalline zinc oxide material. The observed XRD peaks at 2θ = 31.860, 34.487, 36.374, 47.551, 56.593, 62.913, and 68.016 correspond to the respective hkl planes (100), (002), (101), (102), (110), (103), and (112) as shown in Table 1, which confirmed the crystalline zinc oxide nanoparticles with a hexagonal wurtzite phase. High-phase purity and crystallinity are demonstrated by the XRD pattern’s good agreement with the standard pattern of ZnO NPs (JCPDS No. 36-1451). The diffraction peaks are rigid and narrow, indicating the crystalline material. Other crystalline imperfections or impurities cannot be seen, and there is no change in the diffraction peaks.d spacing between polyol-mediated ZnO NPs:

**TABLE 1:** XRD analysis of ZnO NPs.

No.	2θ	Θ	d-Spacing (A^0^)	Intensity (a.u)	(hkl)
1	31.860	15.930	2.8054	3,075.89	100
2	34.487	17.243	2.5976	2,947.53	002
3	36.374	18.187	2.4670	4,072.15	101
4	47.551	23.775	1.9099	1748.55	102
5	56.593	28.296	1.6243	1974.70	110
6	62.913	46.456	1.0622	1883.02	103
7	68.016	34.008	1.3766	1,656.87	112

Bragg’s equation can be used as,

nλ=2d Sin θ,

where λ = 1.54 A^0^ wavelength-X rays, n = order of diffraction (n = 1), d = distance between adjacent layer, and θ **=** glancing angle)

Crystal planes for the cubic system (a = b = c = 4.8152 Å)

$$d_{hkl}=\frac{a}{\sqrt{h2+k2+l2}}.$$

The Scherrer equation,

$$D=\frac{K\;\lambda }{\beta \mathrm{COS}\theta }\;\left(D\leq 200\;nm\right),$$

where D = average crystalline grain size.K = Scherrer constant, (0.68–2.08)β = β′π/180, broadening at FWHM in radians.β’ = full width at half maximum.λ= X-ray wavelength (CuK_α_ = 1.5408 Å)θ = glancing angle.

The average crystalline grain size of the polyol-mediated ZnO NPs is 18.09 nm, measured with the help of the Scherrer equation (Jadhav et al., 2022b).

### 3.4 Field-emission scanning electron microscopy

**FIGURE 4:**
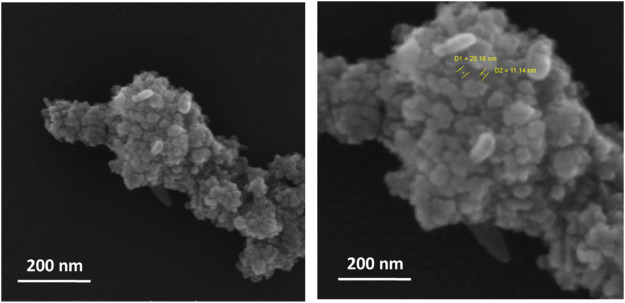
FESEM images of ZnO NPs.

**FIGURE 5:**
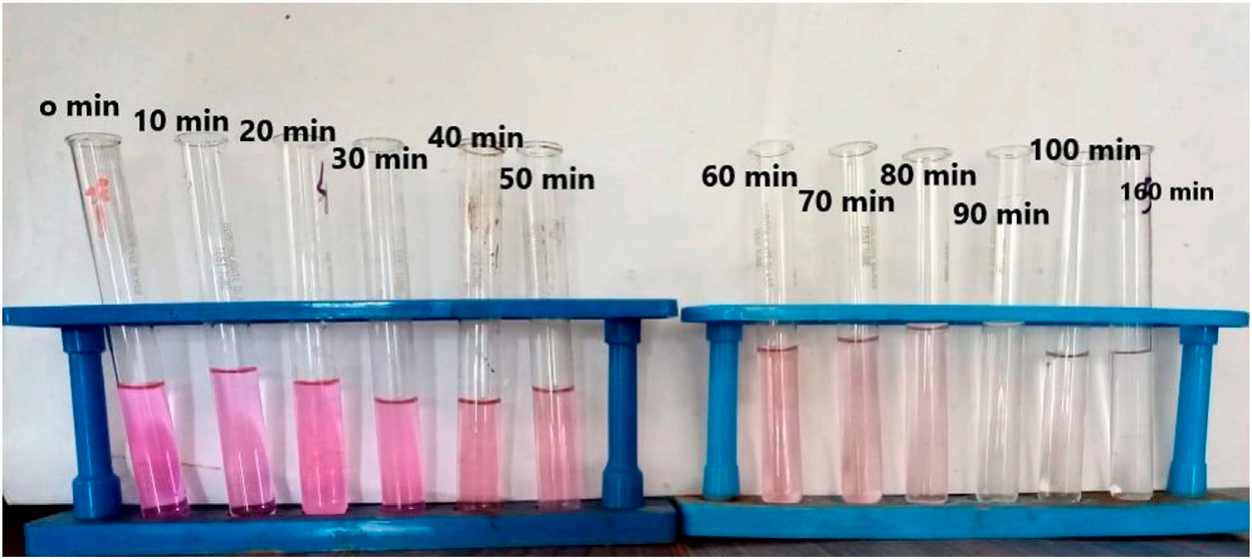
Degradation of dye under UV irradiation.

The FESEM images are essential in providing information on the morphology and size of the synthesized zinc oxide nanoparticles. The spherical and granular nature of the particles indicates their suitability for various applications, including solar cells, sensors, and photocatalysis. The number of aggregates and individual ZnO NPs shown in the images can be used to determine the size distribution of the nanoparticles. The FESEM images confirmed the synthesized polyol-mediated zinc oxide nanoparticles, as shown in Figure 4; the number of aggregates and individual ZnO NPs was shown. The image demonstrates the particles’ spherical, granular, and nanosized nature. XRD analysis shows that the average particle size of ZnO NPs is 18.09 nm (Figure 4), and the FESEM images show that the spherical shapes with a group of aggregated particles are between 10 and 25 nm, as shown in Figure 5. According to the FTIR study, the molecules such as alcoholic compounds cause Zn and O atoms to confirm weak and strong binding peaks.

### 3.5 Photocatalytic degradation

The following steps were used for dye degradation.

*Charge carriers’ generation:* An electron (e^−^) is stimulated from the VB to CB when a semiconductor (ZnO NPs) is exposed to UV light with E ≥ bandgap. When a ZnO NPs is bombarded with enough light energy, it generates electron-hole *(h* ^*+*^ *+ e* ^*-*^ *)* pairs,

$$ZnO+hv\;\left(UV\right)----------------\to \;ZnO\;\left(h^{+}+e^{-}\right).$$

The electron-hole pair is formed when the excitation leaves an ***h*** ^***+***^ in the VB.

*Charge carriers’ trapping:* Electron and hole scavengers capture the (h^+^ + e^−^) pair, thus preventing recombination. h^+^ is an oxidant directly oxidized to dyes or combined with water and an electron donor such as O_2_ and OH^**−**^ to create the HO^**.**^radical (oxidant) as shown in Schemes 1, 2.

$$H_{2}O+h^{+}----------\to \;{OH}^{.}+H^{+},$$

$$h^{+}+{OH}^{-}------------\to \;OH.$$

In contrast, the electron in the CB must be scavenged by an e^−^ acceptor to prevent recombination with the trapped hole. Reactive (O_2_^**.-**^) radical anions are formed when O_2_ is reduced with an e^**-**^. Due to this reaction, other oxidizing species, such as ${HO}_{2}^{.}$ and H_2_O_2_, are generated (Vaseem et al., 2010; Soni et al., 2018). The reactions that create extra OH^**.**^ radicals are described as follows:

$$O_{2}+e^{-}-----\to \;{{2O}_{2}}^{-.}+{2H}^{1}-----\to {{HO}_{2}}^{.}$$

*Charge carriers’ recombination:* (e−h+) pair and trapped carrier recombination may occur in the charge transfer process during this process of heat liberation,

$$ZnO\;\left(e^{-}+h^{+}\right)--------\to \;ZnO+heat.$$

*Photocatalytic degradation of dyes:* The principal photoreactions show that (e^–^ + h^+^) pairs play a crucial role in photocatalytic dye degradation. OH^**−**^, O_2_, and HO_2_^**.**^radicals and photogenerated holes (h+) are highly reactive intermediates that react continuously on the neighboring species, eventually resulting in total degradation of the dye compounds and the oxidative, reductive role of the species, i.e., OH, h^+^, and e^**-**^ in the dye degradation process as shown in Schemes 1, 2.

$${OH}^{-}\;/\;{OH}^{.}+Dye-------------\to \;Degrade+Product.$$

$$H^{+}\;/\;{OH}^{.}+Dye-------------\to \;Degrade+Product,$$

$$O_{2}^{-.}\;/O_{2}^{.}+Dye-------------\to \;Degrade+Product.$$

**FIGURE 6:**
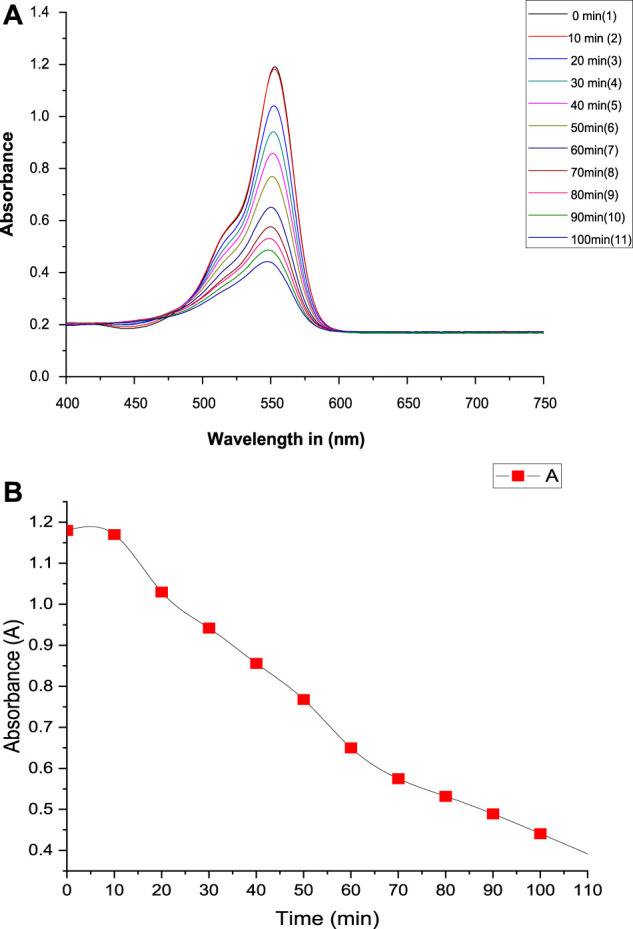
**(A)** Absorbance vs wavelength and **(B)** absorbance vs time (dye degradation plot).

*Experimental photocatalytic dye degradation method:* For the concentrations of ZnO (20 mg/100 mL), the absorption spectra for the model pollutant Rhodamine-B were measured at 10^–5^ M. For the concentration of ZnO NPs, unique to RB, the spectra indicated a wavelength max at 530 nm. The ZnO nanoparticles were left in the dark for 10 min throughout the experiment to be observed, and the dye did not degrade. When ZnO was added to the same stock solution and subjected to UV light radiation, the absorption dramatically altered over 30 min. The absorption at 0 min was more significant for samples with a higher ZnO content than those with a lower ZnO concentration. This is because increased ZnO nanoparticle concentrations will result in more molecules interacting with light waves, increasing absorption. For these samples, the absorption has decreased and is equivalent after 30 min. Continuing the process for 120 min, the absorption was recorded, and the degradation of dye was observed, as shown in Figures 5, 6, and Table 2.

**TABLE 2:** Dye solution absorbance with time (min).

*Sr No.*	Time (min)	**Absorbance (A)**	**The rate constant (K) min** ^**-1**^	Mean value of rate constant (K) min^-1^
*1*	0	1.180	-	8.078 × 10^−3^
*2*	10	1.170	8.510 × 10^−5^	
*3*	20	1.030	6.797 × 10^−3^	
*4*	30	0.942	7.508 × 10^−3^	
*5*	40	0.856	8.024 × 10^−3^	
*6*	50	0.768	8.589 × 10^−3^	
*7*	60	0.650	9.938 × 10^−3^	
*8*	70	0.575	1.026 × 10^−2^	
*9*	80	0.532	9.957 × 10^−3^	
*10*	90	0.489	9.787 × 10^−3^	
*11*	100	0.441	9.842 × 10^−3^	

A greater concentration of ZnO NPs (20 mg/100 mL) caused 96.64% of the dye degradation, but a lower concentration of ZnO (10 mg/100 mL) caused only 83% of the dye degradation after 40 min.

The percentage of degradation was calculated by using,

$$Degradation\;\left(\%\right)=\frac{Co-C}{C}\;x\;100,$$

where C_0_ = initial concentration of dye solution.C = concentration of dye solution at the time (t).

Here, C_0_ (absorbance) = 1.193 and C (absorbance) = 0.04 at t = 160 min.

$$Degradation\;\left(\%\right)=\frac{1.193-0.04}{1.193}\;x\;100,$$

$$Degradation\;\left(\%\right)=96.64\;\%.$$

Since the pseudo-first-order kinetics of photocatalytic degradation of RB dye is followed, the rate constant (k) and half-life time (t_1/2_) were determined using the following equation.

$$K=\frac{1}{t}\;\ln\frac{Co}{C}\;and\;t_{\frac{1}{2}}=\frac{0.693}{K}.$$

The rate constant (K) and t_1/2_ of the dye degradation process are 8.078 × 10^−3^ min^-1^ and 85 min, respectively.

### 3.6 Antibacterial activity

**FIGURE 7:**
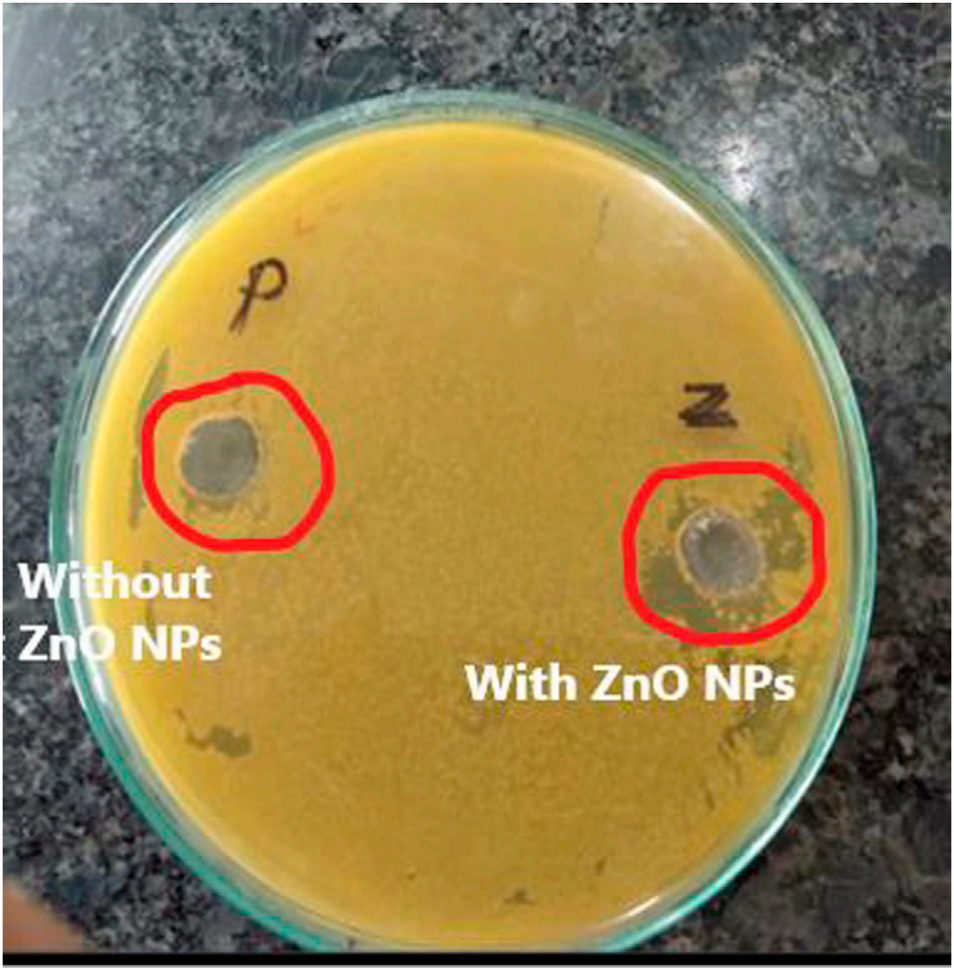
The antibacterial activity of ZnO NPs on *Escherichia coli*.

Agar well diffusion method: The antibacterial activity of the synthesized polyol-mediated zinc oxide nanoparticles was evaluated using the agar well diffusion method against *E. coli*. A bacterial suspension of approximately 10^6^ cells/ml was prepared and spread on a nutrient agar plate. Wells of different sizes ranging from 10 to 60 mm were made on the agar surface using a sterile borer. The ZnO NPs were dissolved in ethanol to prepare stock solutions, and 100 µL of each solution with varying concentrations (20–100 µL) was introduced into the wells (Table 3). The plates were then incubated at 37°C for 24 h. After incubation, the plates were examined for a clear zone of inhibition around the wells, indicating the antibacterial activity of ZnO NPs (Figure 7). The diameter of the zone of inhibition was measured and recorded. The experiment was conducted in triplicate, and the average zone of inhibition diameter was calculated. The results showed that the synthesized ZnO NPs had significant antibacterial activity against *E. coli*, with increasing ZnO NPs resulting in larger inhibition zones. The agar well diffusion method was a simple and effective technique for evaluating the antibacterial activity of the synthesized ZnO NPs (Mendes et al., 2022).

**TABLE 3:** Concentration dependence of the antibacterial activity of ZnO NPs against *Escherichia coli.*

Compound	Concentration	Gram (+) bacteria *Escherichia coli*
ZnO NPs	0.0 μg/mL	ND
	25 μg/mL	9 mm
	50 μg/mL	12 mm
	75 μg/mL	15 mm
	100 μg/mL	18 mm

### 3.7 Antifungal activity

**FIGURE 8:**
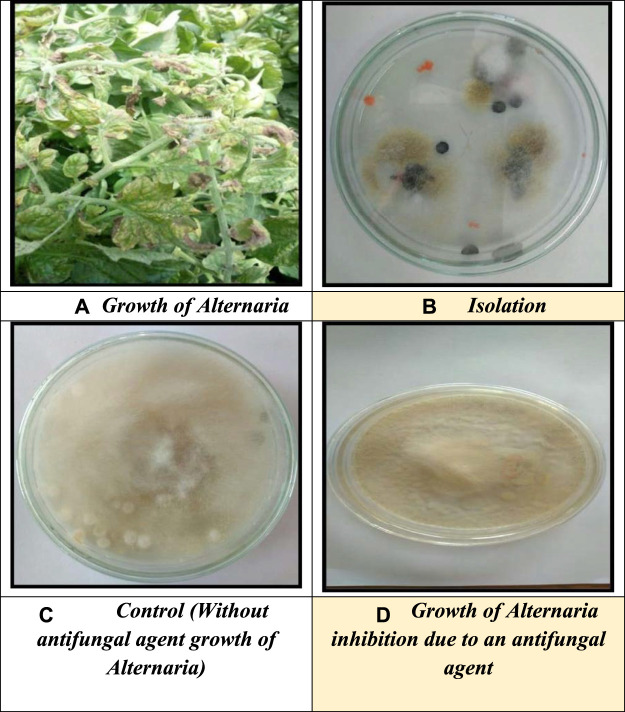
**(A)** Growth of Alternaria, **(B)** isolation, **(C)** control (without antifungal agent growth of Alternaria), and **(D)** growth of Alternaria inhibition due to an antifungal agent.

The colloidal suspension of ZnO nanoparticles was used to study the antifungal activity against the fungal Alternaria, as shown in Figure 8. The altered properties of nanoparticles and their similarity in size compared to naturally occurring biological structures can allow them to readily interact with biomolecules on both the cell surface and within the cell. Antifungal properties of relatively more minor metal oxide nanoparticles have been observed compared to metal nanoparticles. Very few metal oxide nanoparticles’ antifungal properties have been established among these (Alshahrani et al., 2022). In this work, the ability of ZnO nanoparticles was tested against the fungal strain Alternaria, and the medium is agar neutrino. A concentration-dependent growth inhibition effect of ZnO nanoparticles on Alternaria was observed. The minimum inhibitory concentration (MIC) of ZnO nanoparticles for Alternaria was 50.58% (100 μgm/ml). Our study of antifungal activity mechanisms concluded that ZnO nanoparticles penetrate the cells by disrupting the cell membrane, thus inhibiting Alternaria production. These findings reinforce the understanding that ZnO NPs show antifungal properties and should be further explored in biomedical research studies (Salem and Awwad, 2022).

The percent inhibition (L) was calculated by using the formula,

$$L=\frac{C-T}{C},$$

whereC = growth of fungus in the control.T = growth of fungus in treatment (mm).

The result indicates the synthesized nanoparticle has good activity against Alternaria; without an antifungal agent, the growth of Alternaria is 42.5 mm in diameter, and with an antifungal agent, the growth of Alternaria is 29 mm. The percent inhibition was calculated by using the formula,

$$L=\frac{C-T}{C}\;x\;100,$$

whereC = growth of fungus in control.T = growth of fungus in treatment (mm).

$$\begin{array}{c} L=\frac{42.5\;mm-29\;mm}{42.5\;mm}\;x\;100\\ L=\frac{13.5}{42.5}\;x\;100\\ L=0.3176\;x\;100\\ L=31.76\;\% \end{array}.$$

Thus, the percent inhibition is 31.76%.

## 4 Conclusion

This study synthesizes ZnO NPs using ethylene glycol as a chemical reagent and TBAB as a capping agent; the more experimental polyol refluxing approach is a possible substitute for this nanoparticle’s more conventional chemical method. Using ultraviolet-visible spectroscopy, it was further demonstrated that the zinc ions were reduced into ZnO nanoparticles, showing the synthesis of zinc oxide NPs; at 370 nm, the UV spectroscopic absorption peak is located. Furthermore, it was examined by FTIR, XRD, and FESEM. This work evaluated the antibacterial and antifungal activities of the synthesized ZnO NPs in opposition to scientific and conventional concerns, and it also exhibits good photocatalytic activity toward Rhodamine-B. Future studies can focus on optimizing the synthesis process to improve the particle size and morphology, which can enhance the properties of the ZnO NPs. The polyol-mediated ZnO NPs can be a promising candidate for biomedical applications due to their significant antibacterial and antifungal activities.

## Data Availability

The raw data supporting the conclusion of this article will be made available by the authors, without undue reservation.
